# Brief instrument for direct complex functionality assessment: a new ecological tool

**DOI:** 10.3389/fnins.2023.1250188

**Published:** 2023-11-03

**Authors:** Maila Rossato Holz, Renata Kochhann, Patrícia Ferreira da Silva, Maximiliano A. Wilson, Rochele Paz Fonseca

**Affiliations:** ^1^Pontifical Catholic University of Rio Grande do Sul, Porto Alegre, Brazil; ^2^Research Projects Office, Hospital Moinhos de Vento, Porto Alegre, Brazil; ^3^Centre Interdisciplinaire de Recherche en Réadaptation et Intégration Sociale (Cirris) and École des Sciences de La Réadaptation, Faculté de Médecine, Université Laval, Québec, QC, Canada; ^4^Faculty of Medicine, Federal University of Minas Gerais, Belo Horizonte, Brazil

**Keywords:** activities of daily living, neuropsychological testing, aging, Alzheimer disease, mild cognitive impairment

## Abstract

**Background:**

The Direct Assessment of Functional Status (DAFS) is the only instrument validated in Brazil that assesses functionality directly with the patient. However, this clinical tool takes a long time to be administered. This limits its use in hospitals and outpatient clinics that require brief assessment instruments. Additionally, we need to count with a direct assessment because the number of older adults living alone is increasing and we thus lack reliable informants.

**Objective:**

This study aimed to present the development and content validity evidence of a direct complex functionality test for older adults, the Brief Instrument for Direct Functionality Assessment (BIDFA).

**Method:**

A total sample of 30 older adults and eight expert judges took part in the study stages. The BIDFA construction stages were: (1) literature review of functionality instruments; (2) development of seven ecological tasks to evaluate the performance of daily complex activities with the older adults; (3) content analysis by eight expert judges; (4) pilot study with 30 older adults; (5) the ecological analysis of items; (6) focus group analysis; and (7) final version of the BIDFA.

**Results:**

The BIDFA had evidence of content validity with an agreement index of 96.5%. The final version of BIDFA was left with six domains of complex functionality divided into semantic memory and time orientation; shopping skills; executive attention, math and finance skills; organization; planning and procedural memory; and problem-solving. The complex functionality score by BIDFA ranges from 0 to 100 points.

**Conclusion:**

The BIDFA was found to have good content validity by the expert judges and by the ecological analysis of the items by the older adults. The new instrument is expected to help assess the functional status of older adults, in an abbreviated context including complex functionality demands, with a wider range of total and subdomain scores.

## Introduction

Life expectancy is increasing all over the world and, consequently, age-related diseases have also increased. Cognitive changes in older adults have been documented in numerous studies (Drag and Bieliauskas, 2010; Harada et al., 2013). However, when these cognitive changes begin to cause impairments and associated complaints, there is already a greater chance of mild cognitive impairment (MCI; Winblad Palmer et al., 2004; Petersen et al., 2014). MCI involves cognitive impairment in one or more domains with no functional impact and should be understood as a transitory phase target identification for early interventions (Bai et al., 2022; Karimi et al., 2022). As an involution sign or a phenotype marker, dementia such as Alzheimer’s disease (AD) is characterized by one or more cognitive domains with an impact on functionality (Dubois et al., 2016; Jack et al., 2018; Altieri et al., 2021). In this way, functionality determines whether it is a condition of MCI (which may be a pre-dementia) or a dementia itself (American Psychiatric Association, 2014). Dementia usually is a neurodegenerative disease that starts with impairments in instrumental activities of daily living and increases its functional impact until the aggravation of basic activities.

More recent analyses have identified that the extent, severity, type, and management of functional impacts can help in the early detection of AD (Brown et al., 2011). Functionality is the individual’s ability to independently maintain substantial work, financial, academic, affective, social, and domestic activities (Brown et al., 2011). Functionality can be impaired secondary to cognitive changes (Knopman et al., 2003). These changes begin subtly in individuals with MCI (Pereira et al., 2010b; Brown et al., 2011). These changes do not impact independence but begin to show a reduction in cognition with an increase in the individual’s or family’s complaints by observation.

The decline in cognitive functions such as memory or executive functions may be accompanied by a decrease in the effectiveness and quality of performance in functional activities (Harada et al., 2013; Park and Festini, 2017). Thus, the anamnesis interview associated with functionality scales is the main measure for the assessment of functionality in older adults with cognitive complaints. The assessment of both basic and complex (instrumental) functionality is usually performed using scales and questionnaires (Patterson et al., 2001). Among the main scales used for the indirect assessment of functionality are: the Functional Activities Questionnaire (FAQ; Pfeffer et al., 1982), the Informant Questionnaire on Cognitive Decline in the Elderly (IQCODE; Jorm and Jacomb, 1989), the Activities of Daily Living Questionnaire (ADL-Q; Johnson et al., 2004), the Bristol Activities of Daily Living Scale (Bucks et al., 1996), the Disability Assessment for Dementia (Gélinas et al., 1999) and the Bayer Activities of Daily Living Scale (Erzigkeit et al., 2001). In Brazil, the country of the current study, the main ones used with family members of patients with suspected dementia are FAQ, IQCODE, and ADL-Q (Chaves et al., 2011).

However, functionality scales only assess functionality indirectly. As such, they depend on the report of an informant or family member who lives with the patient. These informants may underestimate or overestimate the complaints of impairment or the cognitive impact (Fransen et al., 2018). Conversely, some older adults with cognitive complaints may live alone and not have reliable informants about their functionality. Therefore, the direct assessment of functionality with the individual is more sensitive (Pereira et al., 2008). Direct functionality assessment usually takes place by means of ecological tasks that are lengthy and impractical for hospital environments. Among the main instruments for ecological assessment of functionality for older adults are the Medication Management Ability Assessment (Patterson et al., 2002), the Performance-Based Skills Assessment (Patterson et al., 2001), and the Direct Assessment of Functional Status (DAFS; Loewenstein et al., 1989). These instruments analyze the basic and complex functionality of daily living from domains that simulate activities of daily living (finance, transportation, communication, recreation, planning, etc.). In addition, some ecological functional assessment instruments were also created for conditions such as schizophrenia in which there is an impact on functionality due to psychiatric symptoms. The main instruments found in the literature are the Independent Living Scales (Revheim and Medalia, 2004), the Test of Adaptive Behavior in Schizophrenia (Velligan et al., 2007), and the virtual shopping task (Greenwood et al., 2016). Both ecological instruments created for older adults and those created for serious mental health conditions take a long time to be administered and are, thus, of little use in hospitals and outpatient clinical environments. There is a lack of adequate instruments to assess the functionality of older adults in primary care contexts (Karimi et al., 2022).

Among these direct functionality assessment instruments, only the Direct Assessment of Functional Status (DAFS) is validated in the Brazilian context (Pereira et al., 2008; Fransen et al., 2018). The DAFS assesses time orientation, communication, money-handling skills, shopping skills, dressing/personal hygiene, and eating (Loewenstein et al., 1989). However, the stimuli are time-consuming with a long session of assessment, requiring several materials and assessing, simultaneously, basic and complex activities. There is currently no specific ecological screening instrument to assess the instrumental (complex) functionality of older adults in Brazil. The screening assessment of functionality still follows the models of scales and questionnaires (indirect assessment). Since AD is a progressive disease that initially affects the complex activities of daily living, a direct ecological screening instrument would allow for a more accurate identification of the disease. Therefore, the objective of this study was to present the development process and content validity evidence of the Brief Instrument for Direct Functionality Assessment (BIDFA) for older adults [in Brazilian Portuguese *Instrumento Breve de Avaliação da Funcionalidade direta (IBAF-d)*]. This instrument aims to assess by means of a screening the direct and complex functionality of older adults. It is designed to rapidly complement the cognitive assessment and to contribute to differentiate MCI and dementia due to AD clinical conditions. The BIDFA is intended to be used in hospital and outpatient and inpatient settings in an empirical way to measure functionality, without relying on external perceptions or self-report.

## Methods

### Sample

**Figure 1:**
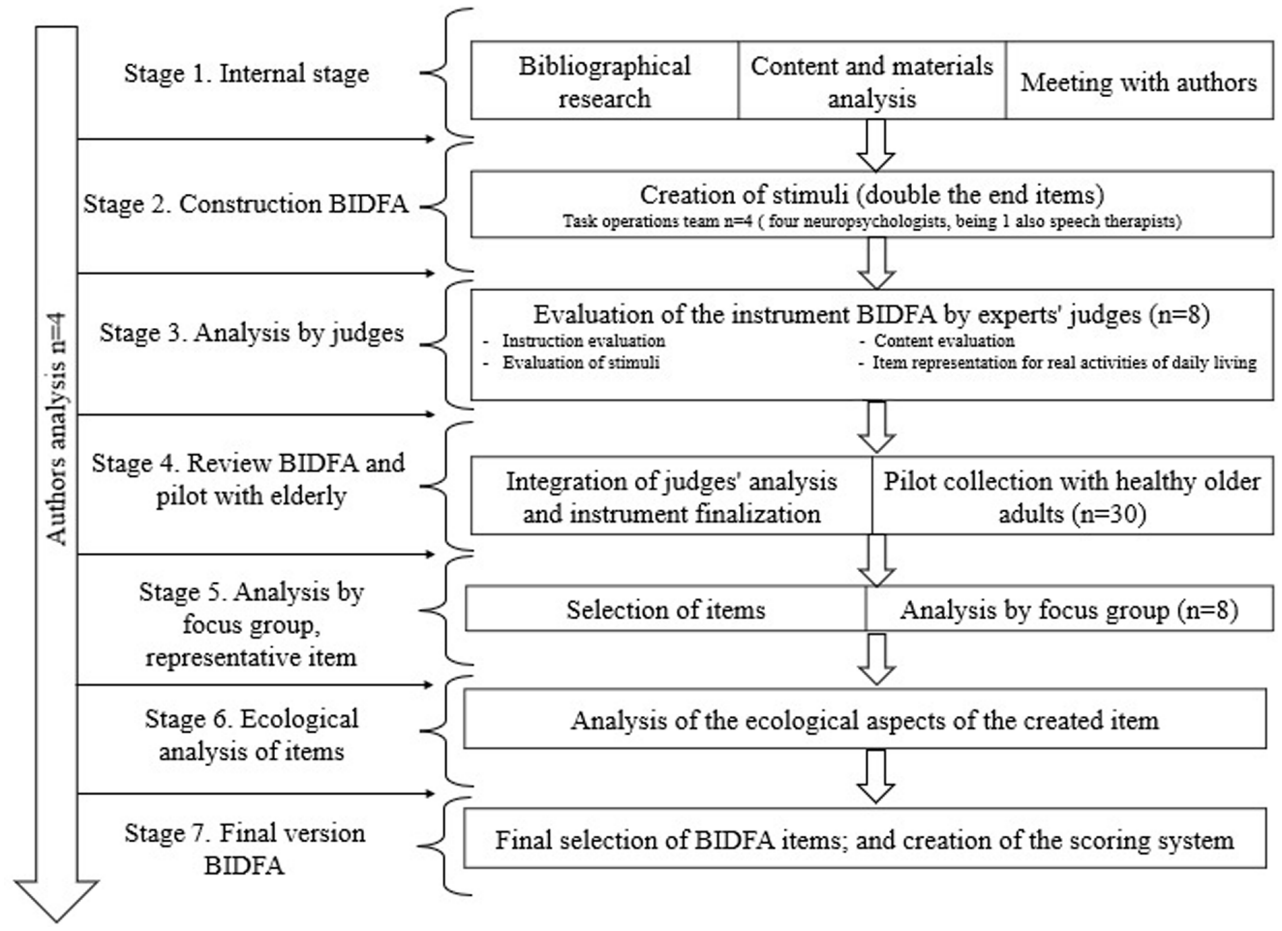
Flowchart depicting each stage of the BIDFA construction process.

The total sample was divided into expert judges and pilot judges in healthy elderly adults. Eight expert judges and 30 older adults participated in the study. The process of creation of the BIDFA took place in seven stages: (1) the internal stage of instrument creation; (2) the construction of the BIDFA instrument; (3) the analysis by expert judges; (4) the review of the BIDFA instrument and pilot study with healthy older adults; (5) the selection of items and focus group analysis; (6) the analysis of how representative the activity is for older adults; and (7) the final instrument and creation of scores. The instrument construction flowchart can be seen in Figure 1.

The analysis of judges (step 3) was performed by two neuropsychologists, two neurologists, two occupational therapists, one speech-language pathologist, and one linguist. The reanalysis performed by the expert judges (step 6) was performed by eight professionals, with two neuropsychologists, one neurologist, two speech-language pathologists, and three psychiatrists. Table 1 presents the areas of expertise of the judges selected for the two stages of the BIDFA assessment.

**Table 1:** Expert judges of the analysis and focus group analysis of the BIDFA.

Judge	Academic background	Level of education
Stage 3 of the BIDFA – experts’ judges		
Judge 1	Neuropsychology	Master’s in Neuroscience
Judge 2	Neuropsychology	Master’s in Psychopharmacology
Judge 3	Neurologist	PhD in Medicine
Judge 4	Neurologist	PhD in Genetics and Molecular Biology
Judge 5	Speech therapist	PhD in Medicine
Judge 6	Linguist	PhD in Linguistics (English)
Judge 7	Occupational therapist	Master in Psychobiology
Judge 8	Occupational therapist	Expert in Neuropsychology
Stage 5 of the BIDFA – focus group		
Judge 1	Psychology	Expert in Neuropsychology
Judge 2	Psychology	PhD student Medical Sciences
Judge 3	Speech therapist	Expert in Neuropsychology
Judge 4	Speech therapist	Expert in Neuropsychology
Judge 5	Neurologist	PhD in Medical Sciences
Judge 6	Psychiatrist	Master’s in Medical Sciences
Judge 7	Psychiatrist	Expert in Neuropsychology
Judge 8	Psychiatrist	PhD in Psychiatry

The expert judges of the BIDFA instrument have the following institutional affiliations: Judges 1, 3, and 4 are from the Federal University of Rio Grande do Sul (UFRGS), Porto Alegre/Brazil; Judges 2 and 5 are from the Federal University of Health Sciences of Porto Alegre (UFCSPA), Porto Alegre/Brazil; Judge 6 is from the Pontifical Catholic University of Rio Grande Do Sul (PUCRS), Porto Alegre/Brazil; Judge 7 is from the Federal University of São Paulo, São Paulo/Brazil; and Judge 8 is from the Federal University of Rio de Janeiro, Rio de Janeiro/ Brazil. The institutional affiliations of the focus group judges are: Judge 1, Unisinos University, São Leopoldo/Brazil; Judge 2, Moinhos de Vento Hospital, Porto Alegre/Brazil; Judges 3, 4, and 6, Pontifical Catholic University of Rio Grande Do Sul (PUCRS), Porto Alegre/Brazil; Judges 5 and 7, Federal University of Rio Grande do Sul (UFRGS), Porto Alegre/Brazil; Judge 8, Federal University of Health Sciences of Porto Alegre (UFCSPA), Porto Alegre/Brazil.

### Procedures and instrument

The construction of the BIDFA followed that of two clinical tools: (1) the Detection Test for Language Impairments in Adults and the Aged (DTLA; Macoir et al., 2017) and (2) the DAFS (Loewenstein et al., 1989). Both authors of these original instruments approved the creation of the BIDFA based on these two clinical trials. The present study was approved by the Research Ethics Committee of the Pontifical Catholic University of Rio Grande do Sul (protocol CAAE 40285820.3.1001.5336) and followed the ethical procedures for research with human beings.

*Stage 1. Internal stage of instrument creation.* The construction of the instrument started with a literature review using the keywords (functionality OR functional OR directed AND screening OR abbreviated OR brief OR brief assessment OR brief task OR brief test OR ecological OR ecological test AND aging OR Alzheimer OR Alzheimer disease OR dementia) to verify the existing instruments of direct functionality for adults and older adults. In the second stage, we verified the components of complex instrumental functionality within each instrument. From this analysis, we created the macrostructure of the main functional domains that should be included in the assessment of the complex functionality of daily life.

*Stage 2. Construction of the BIDFA instrument.* The macrodomains that compose the macrostructure of the BIDFA were selected. The assessment of instrumental functionality should have temporal and spatial orientation, shopping skills (including memory and recognition), numerical calculation skills (finance), organization (tasks involving steps), planning, and problem-solving. Afterward, the stimuli were created based on these macrodomains of functionality. At this stage, twice as many stimuli were created as desired at the end of the instrument. The idea was to choose the items that were better in the pilot stage.

*Stage 3. Analysis by expert judges.* The judges’ analysis was performed based on a protocol to analyze the instructions, the stimuli, the cognitive components involved, and how much the item was representative of a real activity for older adults. The judges answered a questionnaire about how clear the instruction was and how much they agreed with the stimuli of the instrument. Later, in the questionnaire, the judges were asked to assess which cognitive components were involved in that stimulus. At the end, the judges were asked to rate from 0 to 10 how representative the item was of an activity of daily living for older adults.

*Stage 4. Review BIDFA and pilot with healthy older adults.* After the suggestions made by the judges, the first version of the BIDFA was revised before the beginning of data collection. Subsequently, data collection was carried out with 30 older adults with the first version of the BIDFA (which had twice as many stimuli). The older adults in the pilot sample were volunteers recruited from the community. This sample size was included following the recommendation of Johanson and Brooks (2010).

*Stage 5. Selection of items, analysis by focus group.* After collecting data with the sample of older elderly people, a comparison was made between the duplicated items to select those that were more stable and with less error variability. We analyzed and selected the stimuli by means of a focus group. The evaluation was carried out in a presentation format with suggestions for instructions, items, and possible modifications. Subsequently, we performed the analysis of the evaluation of the sample of older adults in relation to the items.

*Stage 6. Analysis of how representative the activity is for the elderly.* During the pilot application, the participants were asked to give a score of one to four points (1 – Rare; 2 – Sometimes; 3 – Frequent; and 4 – Very frequent); and a score from 0 to 10 for how familiar they were with that activity. These items were based on and taken from the protocol applied in the Preventive Intervention Program for Stimulating Executive Functions (EF) in elementary school children (PENcE; de Cardoso et al., 2017).

*Stage 7. Final instrument and creation of scores.* After the focus group analysis, the final items were selected and the final version of the BIDFA was obtained. Subsequently, the final score of the instrument was created following the procedure used in similar tasks, namely the DTLA (Macoir et al., 2017) and the DAFS (Loewenstein et al., 1989). The score ranges from 0 to 100 points.

### Data analysis

We calculated the percentage of agreement between judges for the familiarity with the items by the sample of older adult pilots, and for the frequency with which the individual performs the activity in his daily life. This percentage was calculated from the number of participants who agreed divided by the total number of participants and, subsequently, multiplied by 100 (Alexandre and Coluci, 2011).

The final selection of tasks after the pilot collection with healthy older adults was performed by assigning items 0 or 1. Secondly, the responses were analyzed by taking the mean and standard deviation and selecting the task with the closest score equal to 1. The items selected for the final version were those that showed greater stability in the sample. The verbal tasks of “Planning and procedural memory” and “Problem Solving” were evaluated based on the frequency of verbal responses noted. This model of selection of the BIDFA final items followed the model of Macoir et al. (2017). In stage 5, the content validity index (CVI) was calculated to determine the level of agreement in the pilot sample (Alexandre and Coluci, 2011). This score allows for the analysis of agreement for an item based on the Likert scale. The CVI was calculated from the items scored 3 (Frequent) and 4 (Very frequent) divided by the number of responses.

## Results

*Stage 1. Internal stage of instrument creation.* A search was carried out on PubMed with the keywords to verify the instruments of direct functionality and 92,932 articles were found. Two filters were placed in the search, the first for analysis of the last 5 years and the second for 65 years or more, and 14,629 articles were selected. In this review, we found 17 instruments of direct functionality that were reviewed. When analyzing the instruments of direct functionality, the objective was to analyze which components of complex activity were used in the instruments. In this review, the following domains of instrumental functionality predominated: orientation, communication (start a conversation, social skills, social adjustment), money management (finance), house management (house organization, shopping), transport, planning, and medication administration. Tasks involved simulations (face-to-face or virtual), role-plays, or performance of an activity.

*Stage 2. Construction of the BIDFA instrument.* The instrument was initially divided into six major domains of complex activities of daily living. Initially, twice as many items were created for the pilot and selection from the first data collection with older adults. The domains and tasks created are as follows. (1) Time orientation: task 1 – six temporal orientation items were created from Brazilian national holidays to analyze which one occurs first; task 2 – two analog clocks and two digital clocks to identify which time is earlier and later. (2) Shopping skills: task 1–10 items are presented for the individual to buy at a home, furniture, and construction store. Items were selected based on the frequency of word frequency in Portuguese.^1^ There were three high-frequency items (sieve, knife, and board), three medium-frequency items (hose, broom, and pan), and four low-frequency items (grill, tray, watering can, and vase); task 2 – free recall of shopping items and recognition task from a store shelf with items (with 20 distractors and the 10 target items). (3) Executive attention and math and finance skills: task 1 – a bank statement was presented, asking participants to verify whether certain purchases were possible. Four items were created. In this activity, participants performed the calculations using the sheet; task 2 – four items were created for mental calculations. (4) Organization: Two tasks were created for writing messages. The first is a happy birthday message to a friend. The second is a happy New Year’s message. (5) Planning and procedural memory: four tasks were created requesting the steps to carry out an activity (Medical appointment, dentist appointment, going to the market, and buying medicine). (6) Problem-solving: Four items were created to solve a daily life problem (a burst pipe, new address, shower problem, lack of light). The number of items created for each domain can be seen in Table 2.

**Table 2:** Analysis of representativeness of functional activity in BIDFA and content validity index in a pilot sample (*n* = 30 older adults).

Functional domain	% Representative	Content validity index
Semantic memory and time orientation	86.66%	26 older adults: 4 (Very frequent) 4 older adults: 3 (Frequent) CVI = 1
Shopping skills	93.33%	28 older adults: 4 (Very frequent) 2 older adults: 3 (Frequent) CVI = 1
Executive attention and math and finance skills	100%	30 older adults: 4 (Very frequent) CVI = 1
Organization	90%	27 older adults: 4 (Very frequent) 3 older adults: 3 (Frequent) CVI = 1
Planning and procedural memory	100%	30 older adults: 4 (Very frequent) CVI = 1
Problem-solving	100%	30 older adults: 4 (Very frequent) CVI = 1

*Stage 3. Content analysis by expert judges.* The expert judges showed an agreement index for the BIDFA of 96.50%. The domain with the lowest agreement was “Planning and procedural memory” with 10.82%. It was suggested that the “Organization,” “Planning and procedural memory,” and “Problem-solving” scores be given in the pilot in a descriptive way for the *a posteriori* creation of the final scoring items. The expert judges considered that the total BIDFA had a 92.8% representation of complex activities of daily living. The least representative tasks were “Planning and procedural memory” with 8.91% and “Organization” with 12.49%. All other domains had a 100% agreement among the judges for representativeness in relation to instrumental activities of daily living.

*Stage 4. Review BIDFA and pilot with healthy older adults.* Small suggestions in light of the expert judges’ instructions and changes were incorporated before the pilot. After these adjustments, the preliminary version of the BIDFA was administered to 30 healthy older adults over 55 years of age. All participants had Portuguese as their native language. Data collection took place together with the pilot of the Executive Function Screening (in Portuguese: Triagem das Funções Executivas – TFE) instrument, following the same inclusion and exclusion criteria (Holz, Kochhann, Wilson and Fonseca *in press*). Their mean age was 67 years (SD = 9.58, range = 55–85), their mean level of education was 13 years (SD = 5.17, range = 4–24), and the gender distribution was 5 males and 25 females. A self-report questionnaire was applied to rule out traumatic brain injury and neurological, psychiatric, and other clinical conditions that could impair cognitive performance. All participants were tested in individual rooms with a quiet environment in their homes after they signed the informed consent form. Tasks were administered without any time restrictions.

*Stage 5. Selection of items, analysis by focus group, and analysis.* The selection of items was based on the DTLA (Macoir et al., 2017). Therefore, the selection of the final stimuli was carried out based on the best scores obtained during the pilot with the 30 older adults. The items with the highest success rate for each task were as follows. (1) Time orientation: stimulus one 100%, stimulus two 100%, stimulus three 100%, and stimulus four 100%. (2) Shopping skills: for stimulus one, the participants remembered 3 words (SD = 1.76), and for recognition, the older adults recognized 6.93 items (SD = 2.08). (3) Executive attention and math and finance skills: stimulus one 96% (SD = 0.18), stimulus two 96% (SD = 0.18), stimulus three 93% (SD = 0.26), and stimulus four 93% (SD = 0.26). (4) Organization: stimulus one 80% (SD = 0.68). (5) Planning and procedural memory: stimulus one 68% (SD = 0.62) and stimulus two 72% (SD = 0.47). (6) Problem-solving: stimulus one 100% and stimulus two 96% (SD = 0.26).

The analysis by focus group was carried out based on a meeting to present the final version of BIDFA. Domain 1 (Time orientation) was analyzed by the judges and criticized for only assessing temporal orientation. There was a consensus that the item assessed concurrent semantic memory. Therefore, the domain was renamed as “Semantic memory with time orientation.” (2) One of the stimuli selected for the “Problem-solving” domain was replaced by another one from the pilot. This is because the stimulus selected was biased for individuals who drove and had a driving license. As the instrument is for analyzing possible functional changes, an item that did not involve driving was sought. Therefore, the item “new address” (96%, SD = 0.19) was replaced by the third highest rated “lack of light” (93%, SD = 0.26).

*Stage 6. How representative the activity is for the older adults.* Table 2 presents the analysis of the percentage of the sample of pilot participants and the CVI. This analysis was performed to identify the extent to which the BIDFA domains were representative of instrumental activities of daily living. According to Alexandre and Coluci (2011), the validity of new instruments must have a CVI greater than 0.9. All of the items showed a CVI of 1.

*Stage 7. Final instrument and creation of scores.* The final version of BIDFA is presented in Portuguese in the Supplementary material. The maximum score is 100 points, with each domain having a maximum score of 16 points, except for “Shopping skills” which has a maximum score of 20 points. The estimated administration time is from 10 to 15 min. The construction from the macrostructure to the pilot stimuli, the final selection and the score calculation are presented in Table 3.

**Table 3:** Pilot construction, final version, and BIDFA score.

Functional domain	Pilot stimulus	Final selection	Scoring
Semantic memory and time orientation	Task 1 – Orientation (6 items) Task 2 – Clock (4 items)	Task 1: 2 items Task 2: 2 items	4 points/item
Time orientation			16 points
Shopping skills	Task 1 – Evocation (10 items) Task 2 – Recognition (30 items, 10 target)	Tasks 1 and 2: remained the same	10 points/item
Shopping skills			20 points
Executive attention and math and finance skills	Task 1 – Written calculation (4 items) Task 2 – Mental calculation (4 items)	Task 1: 2 items Task 2: 2 items	4 points/item
Executive attention and math and finance skills			16 points
Organization	Task – Massage (2 written items)	Task: 1 written item.	4 points/item
Organization			16 points
Planning and procedural memory	Task – Planning in stages (4 items)	Task: 2 items	8 points/item
Planning and procedural memory			16 points
Problem-solving	Task – Problems to solve (4 items)	Task: 2 items	8 points/item
Problem-solving			16 points
Total BIDFA			**100 points**

The final score for “Organization” was performed later from the analysis of the pilot sample’s responses. The response frequency of the pilot sample was: 100% of older adults said congratulations/happy birthday; 98% positive nouns for the birthday (health, joy, peace, hope, love, travel, and success); 93% wished the person something (I wish you, that you have, these are my wishes); and 78% included a closing signature (kisses, hugs, and affection). Thus, 53% of the sample gave four categories of responses, 38% three categories, 9% two categories, and 0% one category or no category.

## Discussion

This study aimed to present the construction and content validity of the Brief Instrument for Direct Functionality Assessment [in Brazilian Portuguese *Instrumento Breve de Avaliação da Funcionalidade direta (IBAF-d)*]. The construction of the instrument followed the method and technical rigor of creating and adapting neuropsychological instruments (Casarin et al., 2014; Macoir et al., 2017). It has been adapted and updated from the original version of the Directed Assessment Functional Status (Loewenstein et al., 1989; Pereira et al., 2008). As a complementary clinical tool in an abbreviated context, although DAFS assesses both basic and complex functionality, BIDFA assesses only complex (instrumental) functionality of daily life. The construction of the domains was carried out based on a literature review and six domains were created: (1) Semantic memory and time orientation; (2) Shopping skills; (3) Executive attention and math and finance skills; (4) Organization; (5) Planning and procedural memory; (6) Problem-solving. The agreement among expert judges was 96.5%.

The pilot assessment was performed with healthy older adults. The stimuli selected for the final version were based on the items with the highest percentage of correct answers. The BIDFA total score ranges from 0 to 100. All domains score a maximum of 16 points, except for the shopping skills domain which scores a maximum of 20 points (it contains recall and recognition). The BIDFA has a total score of 100 points, as do the DTLA (Macoir et al., 2017) and the DAFS (Loewenstein et al., 1989; Pereira et al., 2010a). The analysis showed that the instrument was representative of activities of daily living in the sample of older adults. Pilot studies for item-based performance with the target elderly participants, and specific content analysis in relation to the ecological level and daily demands are scarce.

This study has some limitations. The first is that the pilot sample is not representative enough for the instrument to be generalizable to the clinical population. The second limitation is that there is still no sample (healthy and clinical) to identify whether the BIDFA can be a useful tool for assessing the complex functionality of daily life and to discriminate between healthy older adults and elderly persons with declining health. The third limitation is that there is no standardization of what complex activities of daily living are and therefore an empirical review of existing instruments was used for decision-making. Lastly, the BIDFA may not be accurate enough to differentiate between MCI and mild AD due to the subtlety of functionality progression.

The assessment of direct functionality is unusual in the neuropsychological clinic due to the number of objects needed and the duration of time. Therefore, the assessment of functionality is based on indirect instruments that are adapted to the anamnesis and to older adults who live alone. Indirect instruments such as the FAQ, the IQCODE, and the ADL-Q analyze functionality with informants and family members (Chaves et al., 2011). However, it has been observed that due to the increase in life expectancy, the number of elderly people who live alone is also increasing. In developed countries, data show that 25% of elderly people live alone in Canada (Lee and Edmonston, 2019), and 30 to 38% in the United States, England, and France (Reher and Requena, 2018). In Brazil, the percentage of older adults living alone is 15.3% (Negrini et al., 2018). Therefore, the BIDFA is a useful tool in light of the increase in life expectancy and, consequently, the increase in diseases such as AD (Boff et al., 2015). It can be used for primary care of the elderly due to its quick administration (Bai et al., 2022; Karimi et al., 2022). The ecological validity of the BIDFA was studied through content validity performed by expert judges and the target audience, thus making it a clinical task with greater representation.

Most existing direct functionality instruments are based on instrumental and basic activities. However, the BIDFA intends to identify changes in functionality early, thus, the analysis of instrumental functionality is direct. However, some aspects are limited in the instrument such as the analysis of aspects of socialization. Therefore, it is suggested that the BIDFA can always be applied when considering the anamnesis, the aspects of neuropsychiatric and clinical symptoms, and the functionality that can be compromised by factors such as depression, anxiety, and stress (Dubois et al., 2014; Jack et al., 2018). This is because elderly people with neuropsychiatric disorders may present impairment in instrumental activities of daily living (Russo et al., 2007).

The BIDFA test is a screening instrument for the complex functionality of daily living. This instrument is expected to be useful for health professionals to identify as early as possible, based on quantitative data, possible changes in instrumental functionality in the elderly. The main applicability of this new instrument is the identification of the extent and severity of functional problems, helping in the early diagnosis of cases with suspected dementia such as mild AD (Brown et al., 2011). It is also expected that the BIDFA can contribute to estimating the prognosis and creating earlier intervention programs to help elderly patients be more integrated into their family and society groups with daily cognitively demanding activities. Future studies with the BIDFA should focus on an analysis of discriminant, convergent, and cut-off validity with clinical samples and normative data. The BIDFA is expected to help assess the functional status of older adults in a quick fashion and supports complex functionality demands with a wider range of total scores and subdomains for differentiating MCI and mild AD.

## Data availability statement

The raw data supporting the conclusions of this article will be made available by the authors, without undue reservation.

## Ethics statement

The studies involving humans were approved by Research Ethics Committee of the Pontifical Catholic University of Rio Grande do Sul. The studies were conducted in accordance with the local legislation and institutional requirements. The participants provided their written informed consent to participate in this study.

## Author contributions

MH, RK, and RF contributed to the conception and design of the study. MH and PS participated actively in the data collection. MH, RK, MW, and RF contributed to the analysis and interpretation of the results. MH drafted the article. All authors contributed to the article and approved the submitted version.

## Footnotes

^1^ http://lexicodoportugues.com/

## References

[ref1] Alexandre N. M. C. Coluci M. Z. O. (2011). Validade de conteúdo nos processos de construção e adaptação de instrumentos de medidas. Ciencia e Saude Coletiva 16, 3061–3068. doi: 10.1590/S1413-81232011000800006, PMID: 21808894

[ref2] Altieri M. Garramone F. Santangelo G. (2021). Functional autonomy in dementia of the Alzheimer's type, mild cognitive impairment, and healthy aging: a meta-analysis. Neurol. Sci. 42, 1773–1783. doi: 10.1007/s10072-021-05142-0, PMID: 33738665

[ref3] American Psychiatric Association. (2014). DSM-5: Manual Diagnóstico e Estatístico de Transtornos Mentais (5a edição). Porto Alegre: Artmed Editora.

[ref4] Bai W. Chen P. Cai H. Zhang Q. Su Z. Cheung T.. (2022). Worldwide prevalence of mild cognitive impairment among community dwellers aged 50 years and older: a meta-analysis and systematic review of epidemiology studies. Age Ageing 51:afac173. doi: 10.1093/ageing/afac173 35977150

[ref5] Boff M. S. Sekyia F. S. de Bottino C. M. (2015). Prevalence of dementia among brazilian population: systematic review Revisão sistemática sobre prevalência de demência entre a população brasileira. Rev. Med. 94, 154–161. doi: 10.11606/issn.1679-9836.v.94i3p154-161

[ref6] Brown P. J. Devanand D. P. Liu X. Caccappolo E. (2011). Functional impairment in elderly patients with mild cognitive impairment and mild Alzheimer disease. Arch. Gen. Psychiatry 68, 617–626. doi: 10.1001/archgenpsychiatry.2011.57, PMID: 21646578 PMC3682408

[ref7] Bucks R. S. Ashworth D. L. Wilcock G. K. Siegfried K. (1996). Assessment of activities of daily living in dementia: development of the Bristol activities of daily living scale. Age Ageing 25, 113–120. doi: 10.1093/ageing/25.2.113 8670538

[ref8] Casarin F. S. Scherer L. de Parente M. A. Ferré P. Côté H. Ska B.. (2014). Bateria de Avaliação da Comunicação Versão Abreviada-Bateria MAC-Breve (MAC-B). São Paulo, Brasil: Pró-Fono.

[ref9] Chaves M. L. F. Godinho C. C. Porto C. S. Mansur L. Carthery-Goulart M. T. Yassuda M.. (2011). Doença de Alzheimer Avaliação cognitiva, comportamental e funcional. Dementia & Neuropsychologia 5, 153–166. doi: 10.1590/S1980-57642011DN05030003, PMID: 29213740 PMC5619475

[ref10] de Cardoso C. O. Dias N. M. Seabra A. G. Fonseca R. P. (2017). Program of neuropsychological stimulation of cognition in students: emphasis on executive functions - development and evidence of content validty. Dementia e Neuropsychologia 11, 88–99. doi: 10.1590/1980-57642016dn11-010013, PMID: 29213498 PMC5619219

[ref11] Drag L. L. Bieliauskas L. A. (2010). Contemporary review 2009: cognitive aging. J. Geriatr. Psychiatry Neurol. 23, 75–93. doi: 10.1177/0891988709358590, PMID: 20101069

[ref12] Dubois B. Feldman H. H. Jacova C. Hampel H. Molinuevo J. L. Blennow K.. (2014). Advancing research diagnostic criteria for Alzheimer’s disease: the IWG-2 criteria. Lancet Neurol. 13, 614–629. doi: 10.1016/S1474-4422(14)70090-0 24849862

[ref13] Dubois B. Hampel H. Feldman H. H. Scheltens P. Aisen P. Andrieu S.. (2016). Preclinical Alzheimer’s disease: definition, natural history, and diagnostic criteria. Alzheimers Dement. 12, 292–323. doi: 10.1016/j.jalz.2016.02.002, PMID: 27012484 PMC6417794

[ref14] Erzigkeit H. Lehfeld H. Peña-Casanova J. Bieber F. Yekrangi-Hartmann C. Rupp M.. (2001). The Bayer-activities of daily living scale (B-ADL): results from a validation study in three European countries. Dement. Geriatr. Cogn. Disord. 12, 348–358. doi: 10.1159/000051280, PMID: 11455136

[ref15] Fransen N. L. Holz M. Pereira A. Fonseca R. P. Kochhann R. (2018). Accuracy of functional performance in healthy elderly subjects, with mild cognitive impairment and Alzheimer’s disease. Trend. Psychol. 26, 1921–1933. doi: 10.9788/TP2018.4-08En

[ref16] Gélinas I. Gauthier L. McIntyre M. Gauthier S. (1999). Development of a functional measure for persons with Alzheimer’s disease: the disability assessment for dementia. Am. J. Occup. Ther. 53, 471–481. doi: 10.5014/ajot.53.5.471 10500855

[ref17] Greenwood K. E. Morris R. Smith V. Jones A. M. Pearman D. Wykes T. (2016). Virtual shopping: A viable alternative to direct assessment of real life function? Schizophr Res. 172, 206–10. doi: 10.1016/j.schres.2016.02.029 26961185

[ref18] Harada C. N. Love M. C. N. Triebel K. (2013). Normal cognitive aging. Clin. Geriatr. Med. 29, 737–752. doi: 10.1016/j.cger.2013.07.002.Normal, PMID: 24094294 PMC4015335

[ref19] Jack C. R. Bennett D. A. Blennow K. Carrillo M. C. Dunn B. Haeberlein S. B.. (2018). NIA-AA research framework: toward a biological definition of Alzheimer’s disease. Alzheimers Dement. 14, 535–562. doi: 10.1016/j.jalz.2018.02.018, PMID: 29653606 PMC5958625

[ref20] Johanson G. Brooks G. (2010). Initial scale development: sample size for pilot studies. Educ. Psycholog. Measure.-EDUC PSYCHOL MEAS. 70, 394–400. doi: 10.1177/0013164409355692

[ref21] Johnson N. Barion A. Rademaker A. Rehkemper G. Weintraub S. (2004). The activities of daily living questionnaire: a validation study in patients with dementia. Alzheimer Dis. Assoc. Disord. 18, 223–230. PMID: 15592135

[ref22] Jorm A. F. Jacomb P. A. (1989). The informant questionnaire on cognitive decline in the elderly (IQCODE): socio-demographic correlates, reliability, validity and some norms. Psychol. Med. 19, 1015–1022. doi: 10.1017/S0033291700005742, PMID: 2594878

[ref23] Karimi L. Mahboub-Ahari A. Jahangiry L. Sadeghi-Bazargani H. Farahbakhsh M. (2022). A systematic review and meta-analysis of studies on screening for mild cognitive impairment in primary healthcare. BMC Psychiatry 22:97. doi: 10.1186/s12888-022-03730-8, PMID: 35139803 PMC8827177

[ref24] Knopman D. S. Boeve B. F. Petersen R. C. (2003). Essentials of the proper diagnoses of mild cognitive impairment, dementia, and major subtypes of dementia. Mayo Clin. Proc. 78, 1290–1308. doi: 10.4065/78.10.1290, PMID: 14531488

[ref25] Lee S. M. Edmonston B. (2019). Living alone among older adults in Canada and the U.S. Healthcare (Switzerland) 7, 1–22. doi: 10.3390/healthcare7020068, PMID: 31067728 PMC6628335

[ref26] Loewenstein D. A. Amigo E. Duara R. Guterman A. Hurwitz D. Berkowitz N.. (1989). A new scale for the assessment of functional status in Alzheimer’s disease and related disorders. J. Gerontol. 44, P114–P121. doi: 10.1093/geronj/44.4.P114 2738312

[ref27] Macoir J. Fossard M. Lefebvre L. Monetta L. Renard A. Tran T. M.. (2017). Detection test for language impairments in adults and the aged - a new screening test for language impairment associated with neurodegenerative diseases: validation and normative data. Am. J. Alzheimers Dis. Other Dement. 32, 382–392. doi: 10.1177/1533317517715905, PMID: 28639484 PMC10852687

[ref28] Negrini E. L. D. do Nascimento C. F. da Silva A. Antunes J. L. F. (2018). Elderly persons who live alone in Brazil and their lifestyle. Revista Brasileira de Geriatria e Gerontologia 21, 523–531. doi: 10.1590/1981-22562018021.180101

[ref29] Park D. C. Festini S. B. (2017). Theories of memory and aging: a look at the past and a glimpse of the future. J. Gerontol.-Series B Psycholog. Sci. Social Sci. 72, 82–90. doi: 10.1093/geronb/gbw066, PMID: 27257229 PMC5156492

[ref30] Patterson T. L. Goldman S. McKibbin C. L. Hughs T. Jeste D. V. (2001). UCSD performance-based skills assessment: development of a new measure of everyday functioning for severely mentally ill adults. Schizophr. Bull. 27, 235–245. doi: 10.1093/oxfordjournals.schbul.a006870 11354591

[ref31] Patterson T. L. Lacro J. McKibbin C. L. Moscona S. Hughs T. Jeste D. V. (2002). Medication management ability assessment: results from a performance-based measure in older outpatients with schizophrenia. J. Clin. Psychopharmacol. 22, 11–19. doi: 10.1097/00004714-200202000-00003, PMID: 11799337

[ref32] Pereira F. S. Oliveira A. M. Diniz B. S. Forlenza O. V. Yassuda M. S. (2010a). Cross-cultural adaptation, reliability and validity of the DAFS-R in a sample of Brazilian older adults. Arch. Clin. Neuropsychol. 25, 335–343. doi: 10.1093/arclin/acq029, PMID: 20484096

[ref33] Pereira F. S. Yassuda M. S. Oliveira A. Diniz B. Radanovic M. Talib L.. (2010b). Profiles of functional deficits in mild cognitive impairment and dementia: benefits from objective measurement. J. Int. Neuropsycholog. Society: JINS 16, 297–305. doi: 10.1017/S1355617709991330, PMID: 20175938

[ref34] Pereira F. S. Yassuda M. S. Oliveira A. M. Forlenza O. V. (2008). Executive dysfunction correlates with impaired functional status in older adults with varying degrees of cognitive impairment. Int. Psychogeriatrics/IPA 20, 1104–1115. doi: 10.1017/S1041610208007631, PMID: 18752698

[ref35] Petersen R. C. Caracciolo B. Brayne C. Gauthier S. Jelic V. Fratiglioni L. (2014). Mild cognitive impairment: a concept in evolution. J. Intern. Med. 275, 214–228. doi: 10.1111/joim.12190, PMID: 24605806 PMC3967548

[ref36] Pfeffer R. I. Kurosaki T. T. Harrah C. H. J. Chance J. M. Filos S. (1982). Measurement of functional activities in older adults in the community. J. Gerontol. 37, 323–329. doi: 10.1093/geronj/37.3.323 7069156

[ref37] Reher D. Requena M. (2018). Living alone in later life: a global perspective. Popul. Dev. Rev. 44, 427–454. doi: 10.1111/padr.12149

[ref38] Revheim N. Medalia A. (2004). The independent living scales as a measure of functional outcome for schizophrenia. Psychiatr. Serv. 55, 1052–1054. doi: 10.1176/appi.ps.55.9.1052 15345767

[ref39] Russo A. Cesari M. Onder G. Zamboni V. Barillaro C. Pahor M.. (2007). Depression and physical function: results from the aging and longevity study in the Sirente geographic area (ilSIRENTE study). J. Geriatr. Psychiatry Neurol. 20, 131–137. doi: 10.1177/0891988707301865, PMID: 17712095

[ref40] Velligan D. I. Diamond P. Glahn D. C. Ritch J. Maples N. Castilho D.. (2007). The reliability and validity of the test of adaptive behavior in schizophrenia TABS. Psychiatry Res. 151, 55–66. doi: 10.1016/j.psychres.2006.10.007 17379319

[ref41] Winblad Palmer K. Kivipelto M. Jelic V. Fratiglioni L. Wahlund L. O. Petersen R. C. (2004). Mild cognitive impairment - beyond controversies, towards a consensus: report of the international working group on mild cognitive impairment. J. Intern. Med. 256, 240–246. doi: 10.1111/j.1365-2796.2004.01380.x, PMID: 15324367

