# Supplementary material for: Brief instrument for direct complex functionality assessment: a new ecological tool
Source: Front Neurosci. 2023 Nov 3;17:1250188. doi: 10.3389/fnins.2023.1250188 (PMC10654966; doi:10.3389/fnins.2023.1250188)

Nome:_____________________________________ Idade:__________ Escolaridade (em anos): __________

INSTRUMENTO BREVE DE AVALIAÇÃO DA FUNCIONALIDADE DIRETA (IBAF-d)

(versão piloto)

(Holz, Kochhann, Wilson, Fonseca, *em produção*)

1. MEMÓRIA SEMÂNTICA COM ORIENTAÇÃO TEMPORAL

Ao se considerar o ano iniciando em janeiro e terminando em dezembro sobre os feriados nacionais responda:

A) Qual feriado vem primeiro: o dia das mães ou o dia dos namorados?

( ) Dia das mães (4 pontos) ( ) Dia dos namorados (0 pontos)

B) Qual feriado vem primeiro: dia dos pais ou dia dos professores?

( ) Dia dos pais (4 pontos) ( ) Dia dos professores (0 pontos)

Em relação ao horário... (mostrar folha de estímulos)

C) Qual dos relógios mostra o horário **mais cedo**?

( ) Relógio 1 (4 pontos) ( ) Relógio 2 (0 pontos) ( ) Relógio 3 (0 pontos)

D) Qual dos relógios mostra o horário **mais tarde**?

( ) Relógio 1 (0 pontos) ( ) Relógio 2 (0 pontos) **( ) Relógio 3** (4 pontos)

TOTAL ORIENTAÇÃO TEMPORAL= _______________/16

2. MEMÓRIA EPISÓDICA IMEDIATA PARA COMPRAS

Você precisará comprar 10 itens numa loja de casa, móveis e construção. Assim é necessário que você repita comigo os itens da lista porque depois terá que recordar eles para fazer as compras. Preste bastante atenção, pois só posso falar cada item UMA VEZ: (somente repetir o item caso ele repita incorretamente)

1 - Faca ( )	6 - Tábua ( )
2 - Panela ( )	7 - Bandeja ( )
3 - Peneira ( )	8 - Regador ( )
4 - Mangueira ( )	9 - Grelha ( )
5 - Jarra ( )	10 - Vassoura ( )

3. HABILIDADE DE ATENÇÃO EXECUTIVA E MATEMÁTICA PARA COMPRAS

Abaixo você tem um extrato da conta bancária de Cassio com as transações dos últimos dias. (mostrar folha de estímulos)

(o indivíduo pode usar a folha de estímulos para ***escrever e fazer*** os cálculos do extrato)

A) É possível Cassio comprar um ar condicionado com valor de R$ 999,00?

( ) Sim (4 pontos) ( ) Não (0 pontos)

B) É possível Cassio comprar um ar condicionado com valor de R$ 999,00 e um espelho de 120,00?

( ) Sim (4 pontos) ( ) Não (0 pontos)

Correção: (B) Sim; (D) Sim.

Vou pedir que você faça alguns cálculos **mentalmente (de cabeça)** para mim:

C) Arthur precisa comprar alguns itens na farmácia. Ele tem 50,00 reais na carteira e as compras deram 33,00. Quanto Arthur deve receber de troco? ______________________ **( ) Correto (4 pontos)** ( ) Incorreto (0 pontos)

D) Patrícia vai a padaria com 200 reais na carteira. Suas compras deram o valor de 145,00 reais. Quanto Patrícia deve receber de troco? ________________________________ **( ) Correto (4 pontos)** ( ) Incorreto (0 pontos)

Correção: (E) 17 reais; (G) 55 reais.

TOTAL HABILDADADE DE ATENÇÃO EXECUTIVA E MATEMÁTICA PARA COMPRAS=______/16

4. HABILIDADES PARA ORGANIZAÇÃO

Item 6. Você quer escrever uma mensagem de feliz aniversário para um amigo seu/ uma amiga sua. O que você escreveria na sua mensagem? (usar folha de estímulos, não informe os itens abaixo)

( ) Parabéns/ feliz aniversário/ felicidades (4 pontos) ( ) Desejar algo a pessoa (te desejo, que tenhas...) (4 pontos)

( ) Substantivos que representam desejos positivos pelo aniversário (saúde, alegria, paz, amor...) (4 pontos)

( ) Finalização (Beijos, com carinho, abraços...) (4 pontos)

TOTAL HABILIDADE PARA ORGANIZAÇÃO= ________/16

5. MEMÓRIA EPISÓDICA E RECONHECIMENTO PARA COMPRAS

Evocação tardia: “Você recorda os itens que você precisa comprar na loja de casa, móveis e construção?”
( ) Faca ( ) Panela ( ) Peneira ( ) Grelha ( ) Vassoura
( ) Mangueira ( ) Jarra ( ) Tábua ( ) Bandeja ( ) Regador Total de itens:_______________/10

Reconhecimento: “Agora você deve identificar todos os itens que precisa comprar na loja de casa, móveis e construção.”
( ) Faca ( ) Panela ( ) Peneira ( ) Grelha ( ) Vassoura ( ) Mangueira ( ) Jarra ( ) Tábua ( ) Bandeja
( ) Regador Total de itens:_______________/10

6. **PLANEJAMENTO E MEMÓRIA PROCEDURAL**

A) Quais são os passos e as etapas necessárias para você realizar uma consulta com um médico? Anote o que o participante falou, e não informe os itens abaixo.

( ) Agendamento, marcação (4 pontos) ( ) Ver endereço/ ir ao local (4 pontos) Total B=________/8

____

____

B) Quais são os passos e as etapas para você ir ao mercado? Anote o que o participante falou, e não informe os itens abaixo.

( ) Fazer a lista/ ver o que falta (4 pontos) ( ) Fazer as compras/ ir ao mercado (4 pontos) Total B=________/8

____

____

TOTAL PLANEJAMENTO E MEMÓRIA PROCEDURAL= ________/16

7. **RESOLUÇÃO DE PROBLEMAS**

A) Um cano na pia da sua cozinha estoura (fura) e começa a sair e vazar água para todos as partes. O que você precisa fazer para resolver esse problema? Anote o que o participante falou, e não informe os itens abaixo.

( ) Fechar o registro da água (4 pontos); ( ) chamar alguém (encanador, zelador, etc.) (4 pontos)

( ) Sair do local/ não resolver o problema (0 pontos)

____

____

B) Faltou luz na sua casa e você precisa ir ao banheiro, mas tem medo de bater nos objetos até chegar a esse cômodo/ peça (ao banheiro). O que você precisa fazer para resolver esse problema? Anote o que o participante falou, e não informe os itens abaixo.

( ) Ascender uma vela/ lanterna/ celular (8 pontos)

( ) Ir para o local direto/ não resolver o problema (0 pontos)

____

____

TOTAL RESOLUÇÃO DE PROBLEMAS=_______/16

**Memória semântica com orientação temporal**	**_____________________/16**
**Atenção executiva e matemática para compras**	**_____________________/16**
**Habilidade para organização**	**_____________________/16**
**Memória episódica (evocação tardia) para compras**	**_____________________/10**
**Reconhecimento para compras**	**_____________________/10**
**Planejamento e memória procedural**	**_____________________/16**
**Resolução de problemas**	**_____________________/16**
**Total IBAFd**	**_____________________/100**

Folhas de estímulos

INSTRUMENTO BREVE DE AVALIAÇÃO DA FUNCIONALIDADE DIRETA (IBAF-d)

1. ORIENTAÇÃO ESPACIAL E TEMPORAL

C)

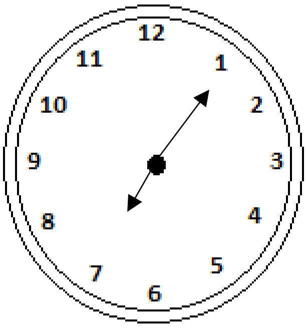

(1)

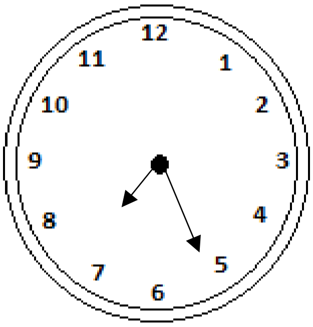

(2)

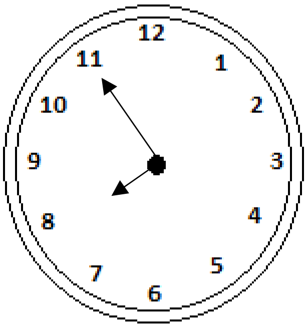

(3)

D)

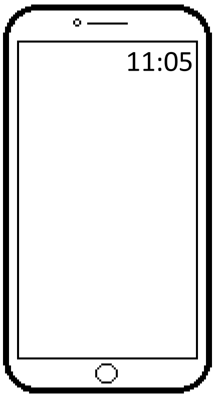

(1)

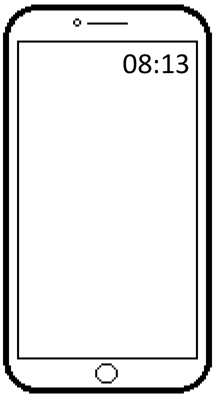

(2)

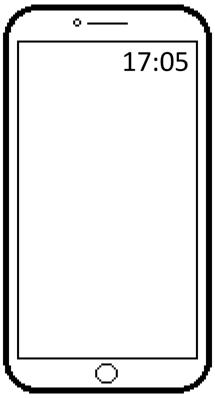

(3)

3. HABILIDADE EM LIDAR COM CÁLCULOS

Data (ano corrente)	Lançamento	Valor (R$)	Saldo (R$)
**01.02**	**SALDO ANTERIOR**		**1.500,00**
01.02	PAGAMENTO FARMÁCIA	- 250,00	
04.02	PAGAMENTO LOJA 1	- 35,00	
04.02	RECEBIMENTO CLIENTE	+ 130,00	
05.02	SUPERMERCADO	- 260,00	
**06.02**	**SALDO CONTA CORRENTE**		**1.085,00**
07.02	LANÇAMENTO FUTURO	+ 115,00	
07.02	LANÇAMENTO FUTURO	- 70,00	

4. HABILIDADES PARA ORGANIZAÇÃO

Item 6. Mensagem de Aniversário

____

____

____

____

____

____

Folhas de estímulos

1. MEMÓRIA EPISÓDICA E RECONHECIMENTO PARA COMPRAS

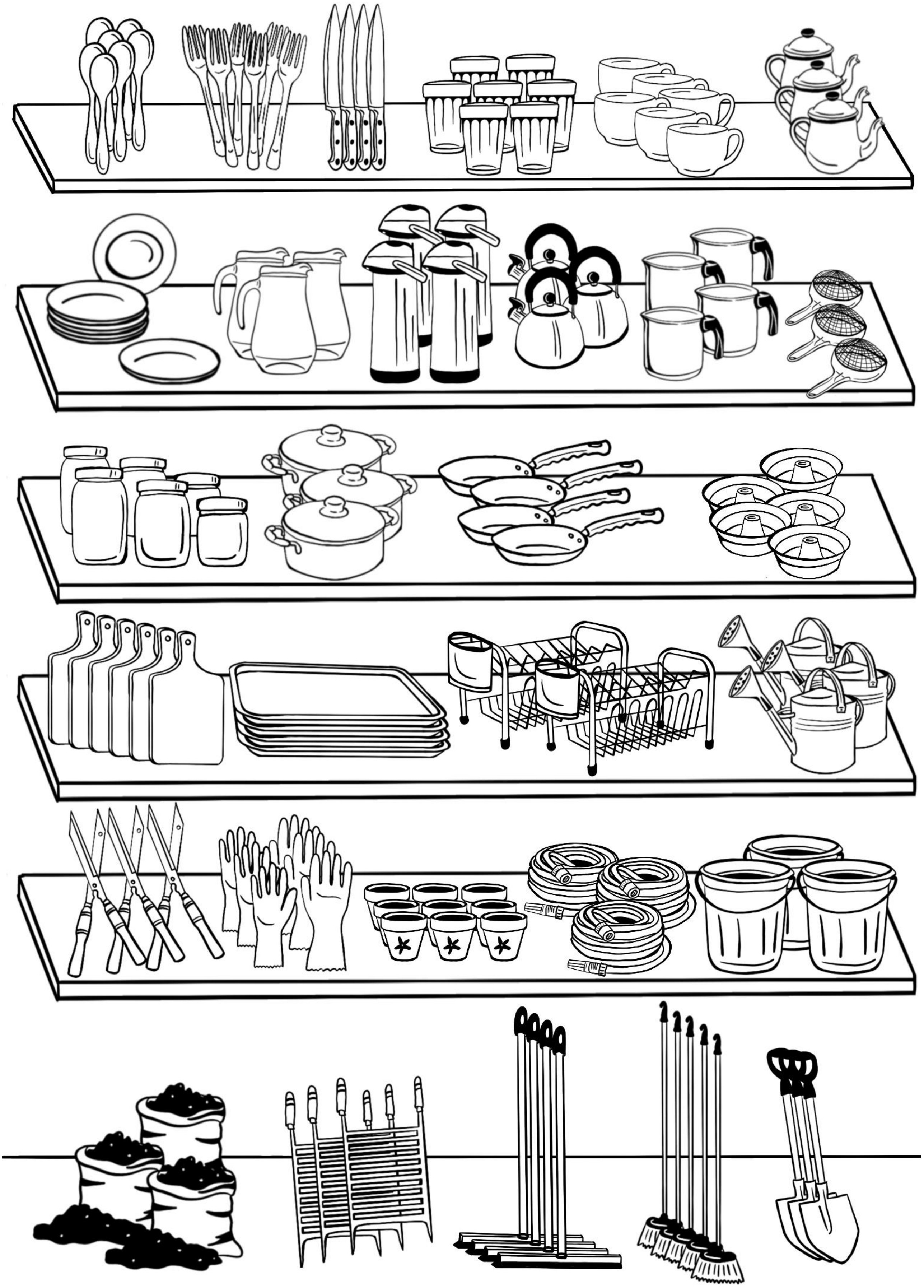

Supplement: Supplementary file 1 [file Data_Sheet_1.PDF]
